# Commentary: First Report of the Italian Registry on Immune-Mediated Congenital Heart Block (Lu.Ne Registry)

**DOI:** 10.3389/fcvm.2020.00083

**Published:** 2020-05-07

**Authors:** Nathalie Costedoat-Chalumeau, Nathalie Morel

**Affiliations:** ^1^AP-HP, Cochin Hospital, Internal Medicine Department, Referral center for Rare Autoimmune and Systemic Diseases, Paris, France; ^2^Université de Paris, CRESS, INSERM, INRA, Paris, France

**Keywords:** neonatal lupus erythematosus, congenital heart block (CHB), fluorinated steroids, lupus (SLE), anti SSA/Ro

We read with great interest the “First Report of the Italian Registry on Immune-Mediated Congenital Heart Block (Lu.Ne Registry)” by Micaela Fredi et al. (1). The authors should be congratulated for the substantial amount of data they have collected about this rare condition. Moreover, they have not only reported 89 cases, but also reviewed the literature and synthesized it in a clear and helpful table (Table 1) that underlines the diversity of treatment strategies, with steroids used at a substantially higher rate in Italy than the other registries (see Table 1). This result confirms the absence of consensus about steroid treatment. It should be noted that their results are otherwise in many aspects consistent with the published large retrospective studies (Table 1). We nonetheless think it useful to discuss this table's numbers concerning incomplete congenital heart block (CHB), for we fear that these figures may be misleading regarding the effects of fluorinated steroids in incomplete CHB.

We would first like to emphasize that the analysis of the literature on this topic is especially difficult for several reasons: (1) some series report cases with no anti-SSA antibodies (2–4); (2) some series do not provide data detailed enough to be analyzed at the individual-patient level for incomplete CHB (2–4); (3) second- and third-degree CHB can be very challenging to distinguish *in utero* as emphasized by Eliasson et al. (2) and Van den Berg et al. (4); (4) some authors define success to include a change from second-degree to alternating back and forth between first and second degree CHB, or from third degree to alternating between second and third degree or stabilizing in second degree, even though the CHB may have been alternating between stages from the beginning and although this may not influence prognosis (2, 5); and (5) last but not least, incomplete CHB may evolve after birth, and success reported at birth is not always confirmed in childhood (2, 4). Moreover, we hypothesize that there is a bias toward more frequent publishing of “successful” cases in smaller series [including in the Italian registry, with successes in its earlier case series (6)].

This being said, we have the following specific comments on this table:

- For the French registry [(7), column Levesque et al.], the table states: regression in 1/13 treated vs. 1/11 untreated, although the figures in the original article were: 1/13 vs. 3/11 untreated fetuses. The three cases included 2 who regressed to first-degree and one to no CHB.
- The analysis of this point in the European/Brazilian registry [(2), column Eliasson et al.] is difficult, perhaps impossible, given the absence of follow-up and the unavailability of antibody status for some cases of incomplete CHB. The table reports that three of seven fetuses with second-degree CHB from mothers positive for anti-SSA and/or anti-SSB treated with fluorinated steroids converted to 1:1 conduction. All three fetuses were indeed in sinus rhythm at birth. However, while one remained in sinus rhythm at 1 year of age, one had reverted to second-degree CHB by 5 years of age, and no information was available for the third. Whether the success rate is 3/7 or 2/7 is thus a question of interpretation. We also note that among the untreated fetuses, antibody status was unknown for 5 of the 8 with second-degree CHB.
- Van den Berg et al. wrote in their original article (4): “*in utero* regression of atrioventricular block (AVB) was observed in three fetuses. Two fetuses with AVB-II° regressed to sinus rhythm (SR) *in utero* and one fetus to AVB-I°. The first fetus, from an SSA seronegative mother, did not receive steroids. The second fetus had been treated with steroids since diagnosis of AVB-II° and converted to SR. The third fetus regressed to AVB-I° spontaneously, whereupon steroids were initiated for the first time. The child progressed to AVB-II° two weeks after birth.” We consider that the mother negative for anti-SSA should not be included, and we would like to note that the last case reverted before steroids (and was potentially even aggravated by them). Van den Berg et al. concluded that they “found no difference in the proportion of AVB-II° progression between steroid-treated and untreated fetuses and observed only an incidental case of AVB regression.” Moreover, they noted that among 21 fetuses diagnosed with AVB-II° (38%) and 35 with AVB-III° (62%), the AVB-II° diagnosis was “revised in 10 cases after reassessment of the echocardiogram by the researchers.” This point emphasizes the difficulty of diagnosing this condition. It is again very difficult to determine the number of cases with regression in the table by Fredi et al., since the original article by Van den Berg et al. does not provide the denominators for treated and untreated cases with anti-SSA. It is in any case most likely fewer than the 42 stated by Fredi et al., since Van den Berg's Figure 2 reports 8 cases of treated first- and second-degree CHB and 12 of untreated second-degree CHB (4).
- Adding up the numbers in this Table 1 seems to show that fluorinated steroids may have reversed second-degree CHB in 15 of 71 treated fetuses, or 21%, including 3/7 from the study by Eliasson et al., 4/13 by Izmirly et al., 1/13 by Levesque et al., 2/14 by Van den Berg, and 5/24 by Fredi et al.; reversals appear to have occurred in 3 of 69 untreated fetuses (4.3%), including 0/8 (Eliasson), 1/8 (Izmirly), 1/11 (Levesque), 1/42 (Van den Berg) and 0/0 (Fredi) (*P* = 0.08). However, when we regroup the cases we consider analyzable (US, French, and Italian) and use the numbers discussed above, we see instead that CHB reverted to first-degree CHB or normal sinus rhythm in 10 of 50 treated fetuses, or 20%, including 4/13 from Izmirly et al., 1/13 from Levesque et al. and 5/24 from Fredi et al.), compared with 4 of 19 (21.1%, including, respectively 1/8, 3/11, and 0/0) untreated fetuses (*P* > 0.99).

In conclusion, this letter shows the difficulty in interpreting the effect of fluorinated steroids and the need for caution before concluding that they may be beneficial in incomplete degree CHB (8, 9). If, as we believe, there is no proof of the usefulness of treatment with fluorinated steroids (and their associated side effects are well-known), it logically follows there is no evidence that routine echocardiographic screening to detect CHB in anti-SSA-positive pregnant women is useful. We have recently discussed this in a viewpoint and concluded that, except in the context of research protocols, overturning the dogma of routine repeated screenings for CHB could save money and health-care staff time and prevent maternal stress without substantial clinical consequences (9).

## Author Contributions

NC-C and NM wrote and reviewed the manuscript.

## Conflict of Interest

The authors declare that the research was conducted in the absence of any commercial or financial relationships that could be construed as a potential conflict of interest.

## References

[1] Fredi M Andreoli L Bacco B Bertero T Bortoluzzi A Breda S. First report of the italian registry on immune-mediated congenital heart block (Lu.Ne Registry). Front Cardiovasc Med. (2019) 6:11. 10.3389/fcvm.2019.00011 30873413 PMC6404544

[2] Eliasson H Sonesson SE Sharland G Granath F Simpson JM Carvalho JS. Isolated atrioventricular block in the fetus: a retrospective, multinational, multicenter study of 175 patients. Circulation. (2011) 124:1919–26. 10.1161/circulationaha.111.041970 21986286

[3] Lopes LM Tavares GM Damiano AP Lopes MA Aiello VD Schultz R. Perinatal outcome of fetal atrioventricular block: one-hundred-sixteen cases from a single institution. Circulation. (2008) 118:1268–75. 10.1161/CIRCULATIONAHA.107.735118 18765396

[4] Van den Berg NW Slieker MG van Beynum IM Bilardo CM de Bruijn D Clur SA. Fluorinated steroids do not improve outcome of isolated atrioventricular block. Int J Cardiol. (2016) 225:167–71. 10.1016/j.ijcard.2016.09.119 27728859

[5] Izmirly PM Saxena A Kim MY Wang D Sahl SK Llanos C. Maternal and fetal factors associated with mortality and morbidity in a multi-racial/ethnic registry of anti-SSA/Ro-associated cardiac neonatal lupus. Circulation. (2011) 124:1927–35. 10.1161/CIRCULATIONAHA.111.033894 21969015 PMC3206147

[6] Ruffatti A Cerutti A Favaro M Del Ross T Calligaro A Hoxha A. Plasmapheresis, intravenous immunoglobulins and bethametasone - a combined protocol to treat autoimmune congenital heart block: a prospective cohort study. Clin Exp Rheumatol. (2016) 34:706–13. 27385463

[7] Levesque K Morel N Maltret A Baron G Masseau A Orquevaux P. Description of 214 cases of autoimmune congenital heart block: results of the French neonatal lupus syndrome. Autoimmun Rev. (2015) 14:1154–60. 10.1016/j.autrev.2015.08.005 26284740

[8] Brucato A Tincani A Fredi M Breda S Ramoni V Morel N. Should we treat congenital heart block with fluorinated corticosteroids? Autoimmun Rev. (2017) 16:1115–8. 10.1016/j.autrev.2017.09.005 28899797

[9] Costedoat-Chalumeau N Morel N Fischer-Betz R Levesque K Maltret A Khamashta M Routine repeated echocardiographic monitoring of fetuses exposed to maternal anti-SSA antibodies: time to question the dogma. Lancet Rheum. (2019) 3:133–94. 10.1016/S2665-9913(19)30069-4 38229394

